# The Development of Severe Neonatal Alloimmune Thrombocytopenia due to Anti-HPA-1a Antibodies Is Correlated to Maternal *ABO* Genotypes

**DOI:** 10.1155/2012/156867

**Published:** 2011-11-02

**Authors:** Maria Therese Ahlen, Anne Husebekk, Mette Kjær Killie, Jens Kjeldsen-Kragh, Martin L. Olsson, Bjørn Skogen

**Affiliations:** ^1^Department of Laboratory Medicine, University Hospital of North Norway, 9038 Tromsø, Norway; ^2^Department of Immunology, Institute of Medical Biology, University of Tromsø, 9037 Tromsø, Norway; ^3^Department of Immunology and Transfusion Medicine, Oslo University Hospital, Ullevål, 0407 Oslo, Norway; ^4^Faculty Division Ullevål University Hospital, University of Oslo, 0407 Oslo, Norway; ^5^Division of Hematology and Transfusion Medicine, Department of Laboratory Medicine, Lund University, SE-221 00 Lund, Sweden

## Abstract

*Background*. Maternal alloantibodies against HPA-1a can cross placenta, opsonize foetal platelets, and induce neonatal alloimmune thrombocytopenia (NAIT). In a study of 100, 448 pregnant women in Norway during 1995–2004, 10.6% of HPA-1a negative women had detectable anti-HPA-1a antibodies. *Design and Methods*. A possible correlation between the maternal ABO blood group phenotype, or underlying genotype, and severe thrombocytopenia in the newborn was investigated. *Results*. We observed that immunized women with blood group O had a lower risk of having a child with severe NAIT than women with group A; 20% with blood group O gave birth to children with severe NAIT, compared to 47% among the blood group A mothers (relative risk 0.43; 95% CI 0.25–0.75). *Conclusion*. The risk of severe neonatal alloimmune thrombocytopenia due to anti-HPA-1a antibodies is correlated to maternal *ABO* types, and this study indicates that the observation is due to genetic properties on the maternal side.

## 1. Introduction

Foetal-maternal incompatibility in the human platelet antigen (HPA)-1 alloantigen system is the most common underlying cause of neonatal alloimmune thrombocytopenia (NAIT), a condition where maternal alloantibodies opsonize foetal platelets during pregnancy and reduce their survival in circulation. The incompatibility is based on a single-nucleotide polymorphism (SNP) which results in a leucine/proline substitution at residue 33 in the *β*3 integrin that constitutes membrane glycoprotein *β*3 [GPIIIa] present on platelets in complex with *α*IIb integrin [GPIIb] [1]. On platelets, the *α*IIb*β*3 [GPIIb/IIIa] is also the major carrier of blood group A antigen [2].

About 10% of HPA-1a negative women who have been pregnant with an HPA-1-incompatible child have detectable HPA-1a antibodies [3]. In several studies, a correlation between maternal antibody level and the severity of thrombocytopenia in the newborn has been shown [4–6]. The alloimmunization is strongly associated with the HLA-DRB3*01 : 01 allele [3, 7, 8]; however, only about 30% of the women with this HLA antigen are immunized. Except for the incompatibility in platelet antigen and the association to HLA, other factors which may influence the immune response to HPA-1a have not been identified.

In the present study, we have examined the maternal ABO blood groups and frequency of HPA-1a-immunization of the women identified in the large prospective screening and intervention study carried out in Norway from 1995 to 2004. We included 152 HPA-1a-immunized women, 146 of whom had altogether 158 HPA-1-incompatible pregnancies in the screening study. The ABO distribution among immunized women was investigated, and the maternal ABO phenotype and *ABO *genotype was correlated to the severity of thrombocytopenia of the newborn.

## 2. Materials and Methods

### 2.1. Patients

Pregnant women were recruited for HPA-1 allotyping from three regions in Norway between December, 1995 and March, 2004 [3]. Samples for routine Rh(D) typing were also used for determining HPA-1 allotype by flow cytometry (anti-CD61 mAb), enzyme-linked immunosorbent assay (ELISA), or polymerase chain reaction (PCR) as previously described [9]. A total of 100,448 pregnant women were typed for the platelet antigen HPA-1a, and 2,111 of those were HPA-1a negative (2.1%). Of these, 1,990 were further tested, and anti-HPA-1a antibodies were detected in 154 women during the pregnancy. In total, 146 of these immunized women underwent 158 HPA-1a-incompatible pregnancies. ABO blood group typing was performed by conventional technique. Genomic typing of HPA-1 (*ITGB3;* rs5918 in dbSNP) and *ABO* in the neonates was performed in samples from cord blood or buccal swabs. For the newborns, the *ABO* genotype was used to predict the ABO blood group. In this context, we have defined ABO incompatibility only as an A_1_ phenotype in the newborn, in blood group O mothers, because individuals with A_2_, and the majority of individuals with B phenotype, express only low levels of corresponding antigens on the surface of platelets [2, 10–12]. Thrombocytopenia was defined as a platelet count ≤150 × 10^9^/L, and severe thrombocytopenia less than 50 × 10^9^/L measured in cord blood and/or capillary blood at birth. Detection of anti-HPA-1a IgG antibodies was performed by flow cytometry and quantified with monoclonal antibody immobilization of platelet antigen assay (MAIPA) [3], by using the anti-CD61 monoclonal antibody clone Y2/51 (Dako, Glostrup, Denmark) for immobilisation of platelet glycoproteins. Women were tested at several time points during the pregnancy, and those with a positive antibody test at any time during the pregnancy were characterized as immunized. Nineteen women were primary immunized during the studied pregnancy, 13 of these were primigravida. All others may have been immunized in connection with a prior pregnancy. Prior affected pregnancies were not excluded as a cause of severe NAIT. The NAIT diagnosis was based on maternal anti-HPA-1a antibodies and HPA-1a antigen incompatibility. Other possible reasons for thrombocytopenia (infection, maternal ITP, etc.) were not registered. Informed consent was provided in accordance with the declaration of Helsinki. The study was approved by the Regional Committee for Medical Research Ethics, North Norway (approval no. P-REK V 13/1995).

### 2.2. ABO Genotyping

*ABO* genotyping was performed by PCR-RFLP analysis to detect six major alleles, *A^1^*, *A^2^*, *B*, *O^1^*/*O^1v^*, and *O^2^* (also known as *A101/A201/B101/O01/O02/O03*) according to the nomenclature used by the Blood Group Antigen Gene Mutation Database, dbRBC [13], and further discrimination between the common *O^1^* and *O^1v^* alleles [*O01/O02*] was performed using primers and reaction conditions as described by Olsson and Chester [14, 15] with some modifications: HotStarTaq polymerase 5 U/*μ*L (QIAGEN, Hilden, Germany) was used with the following cycling programs for both analyses: 95°C 15 min, 10 cycles of 94°C for 10 seconds, 63°C for 30 seconds and 72°C for 30 seconds followed by 25 cycles of 94°C for 10 seconds, 61°C for 30 seconds and 72°C for 30 seconds for samples with 100 ng DNA template. For samples with ~25 ng template and <10 ng DNA template, 1 and 2 extra cycles at each of the annealing temperatures was performed, respectively. Subsequently, digestion with endonucleases and qualitative analyses of product were performed [14, 15]. Ambiguous results were confirmed with selected primer sets from a recently published PCR-ASP method for *ABO* genotyping [16]. For simplicity, only one terminology, dbRBC, will be used throughout this paper. Genotypes are written as *X/X*.

### 2.3. Statistical Analysis

Standard statistical calculations as mean, relative risk, Chi-square test, analysis of variance with Bonferroni's test as post hoc analysis, and plots were performed with computer software SPSS for windows (Statistical Package for the Social Sciences, Version 16.0 SPSS Inc., Chicago, Ill, USA). *P* < 0.05 was considered significant. 

## 3. Results

### 3.1. The ABO Phenotype Distribution of the Immunized Mothers

The ABO phenotype distribution among 154 HPA-1a immunized women was similar to the distribution of the general Norwegian population [17] adjusted to statistics for 2005 (data not shown), indicating that the maternal ABO type does not influence the risk of HPA-1a immunization.

### 3.2. Maternal ABO Blood Group and Risk of Severe NAIT

In 158 HPA-1-incompatible pregnancies with 83 cases of NAIT, there were 54 cases of severe NAIT. The maternal ABO phenotypes were compared to the platelet count in their neonates (Table 1); 46.6% of the immunized women with blood group A gave birth to children with severe NAIT, compared to 20.0% among the immunized mothers with blood group O. The relative risk of NAIT (platelet count ≤150 × 10^9^/L) in the neonates of HPA-1a immunized women with blood group O as compared to blood group A was 0.67 (95% CI 0.48–0.94), whereas the relative risk of severe NAIT (platelet count <50 × 10^9^/L) in the neonates of HPA-1a immunized women with blood group O was 0.43 (95% CI 0.25–0.75) as compared to the neonates of women with blood group A. However, the frequency of moderate NAIT (platelet count 50–150 × 10^9^/L) was not lower among the blood group O mothers. 91% of the immunized women carried the HLA-DRB3*01 : 01 allele. There were no cases of severe NAIT among HLA-DRB3*01 : 01-negative mothers.

### 3.3. ABO Incompatibilities between Mothers and Newborns

To investigate whether the ABO incompatibility between mother and foetus could explain the difference in the frequency of severe NAIT, newborns were *ABO* genotyped as basis for the prediction of their ABO phenotype. One hundred and thirty out of 146 newborns were *ABO* genotyped (restricted by lack of material). For ABO-incompatibility studies, thus only 52 of 60 blood group O mother-child pairings could be included. The fifty-two mothers with blood group O gave birth to 16 A-incompatible (blood group A_1_) and 36 compatible (sixteen blood group O, one B, and five A_2_) children. Four of the 16 A-incompatible pregnancies resulted in a newborn with severe thrombocytopenia compared to 6 of the 36 ABO-compatible pregnancies. This indicates that ABO incompatibility is not the underlying cause of the observed phenomenon reported in the present study.

### 3.4. The ABO Genotype of the Mothers and Platelet Counts in the Newborn

The *ABO* genotype of 143 HPA-1a-immunized women who gave birth to 155 HPA-1a-positive neonates was determined (data not shown). The overall* O* allele frequencies among the immunized women were *O01* 0.56, *O02* 0.42, and *O03* 0.02. Individuals with blood groups A, B, and O were further subgrouped based on genotyping, and thus, the frequencies of newborns with severe NAIT within each subgroup were compared. The cases with maternal blood group AB were excluded for further analysis due to the low number of individuals. Analysis of the platelet counts in newborns of mothers with different *ABO *genotypes revealed that the frequency of newborns with severe NAIT differed (Pearson Chi-square *P* = 0.0036) among the maternal *ABO* genotype groups. 

Among blood group A mothers, the frequency of newborns with severe NAIT was 42% in pregnancies where the mother carried only one *A* allele (*A101* or *A201*), compared to 69% where mothers carried two *A* alleles (relative risk 0.61; 95% CI 0.38–0.98). In pregnancies where the mother had blood group O, the frequency of newborns with severe NAIT was 9%, where the mother did not carry any *O0*2 allele, compared to 27% where the mother carried one or two *O02* alleles; however, this did not reach statistical significance (relative risk 0.33 NS *P* = 0.13). 

**Figure 1:**
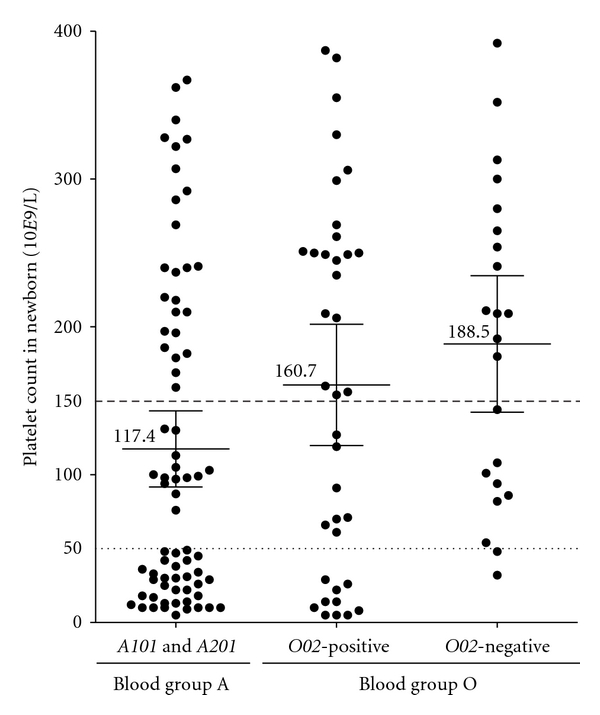
The platelet count at delivery in HPA-1a-positive newborns was grouped according to maternal ABO type (A or O). Platelet count ≤150 × 10^9^/L (dashed line) is defined as NAIT and <50 × 10^9^/L (dotted line) as severe NAIT. The mean platelet counts in the three groups are indicated, with error bars representing 95% CI. Mean platelet counts are significantly different (*P* < 0.019, one-way ANOVA). The mean antibody levels between these groups are not significantly different: 11.6 IU/mL for blood group A mothers, 11.1 IU/mL for *O02*-positive blood group O mothers, and 1.8 IU/mL *O02*-negative blood group O mothers.

Platelet counts in newborns of mothers with blood group A and O are plotted in Figure 1. The mean antibody levels between these groups were not significantly different: 11.6 IU/mL for blood group A mothers, 1.8 IU/mL for O02-negative blood group O mothers, and 11.1 IU/mL for O02-positive blood group O mothers (*P* = 0.18 one-way ANOVA). However, the correlation between the maternal antibody level and platelet count in the newborn for these cases was described in Killie et al. [6].

Among the NAIT cases, defined as platelet count ≤150 × 10^9^/L, the mean platelet count in newborns of homozygous *O01/O01* mothers was higher (83.2 × 10^9^/L) than in the newborns with *O02*-positive mothers with blood group O (43.7 × 10^9^/L) or in newborns of women with blood group A (46.1 × 10^9^/L) (Table 2). Together, these data support our hypothesis that there are genetic properties among the immunized women influencing the risk of severe NAIT in the newborn.

## 4. Discussion

The ABO phenotype distribution among the HPA-1a immunized women is similar to the distribution in the Norwegian population, indicating that the generation of an immune response with antibody synthesis is independent of the ABO blood group of the mother. However, we observed that whereas only 20% of pregnancies among the immunized women with blood group O resulted in severe NAIT in the newborn, 47% of the immunized women with blood group A had newborns with severe thrombocytopenia. A recent retrospective study by Bertrand et al. did not find any significant correlation between the severity of the thrombocytopenia and the *ABO* genotype [18]. As these authors propose, the discrepancy between Bertrands's and our study may be due the retrospective/prospective nature of the studies. We found no indications that the low frequency of severe NAIT in the children of women with blood group O was due to ABO incompatibility between mother and foetus. Additional measurements of maternal anti-RBC IgG antibody in the women with blood group O could have given further information of any influence of potential antibodies directed against the A antigen carried by *α*IIb on platelets. Another hypothesis that could explain the lower frequency of newborns with severe NAIT among the immunized mothers with blood group O, compared to blood group A, is that the *ABO* gene is located close to a gene encoding an immunoregulatory factor with polymorphic variants. In order to approach this question, we compared NAIT to *ABO* genotypes. The allelic differences in the gene encoding the A/B glycosyltransferases are defined by SNPs that changes the amino acid sequence of the enzyme and thereby its glycosylating properties. The *A101*, *A201*, *B101*, *O01*, *O02*, and *O03* alleles all produce transcripts (although *A* transcripts are virtually undetectable in peripheral blood) [19, 20], but the* O01 *and* O02* transcripts both contain a shift in the reading frames that will severely truncate any resulting protein and leave it without enzymatic activity [21]. It is still unclear if these short nonfunctional proteins are expressed at all although it has been suggested [22]. 

The *ABO* genotype frequencies in the Norwegian population are not known, but the *O* allele frequencies observed in immunized women are similar to the frequencies reported for a Swedish population [15], where the *O02* constitutes about 40% of all *O* alleles and *O03* allele is infrequent. This further shows that the generation of an immune response to HPA-1a is independent of ABO blood groups. However, when it comes to development of NAIT, the different risks of severe thrombocytopenia observed in genetic subgroups of blood group A support the hypothesis that a genetic linkage may be involved, rather than the ABO phenotype itself even though the mechanism is still not understood. Although the differences in the *O02-*positive and *O02*-negative subgroups of blood group O do not reach statistical significance, an interesting trend is observed.

Phylogenetic analyses of the *ABO* locus have shown that the *O02* probably is an ancient allelic lineage at the *ABO* locus, separate from the *A101* and *O01* alleles [23]. Therefore, it is interesting to subdivide the blood group O women according to their genotype. The 9q34 chromosomal region, where the *ABO* gene is located [24], contains several loci encoding immune response regulating genes. There is obviously no genetic linkage between the *ABO* [9q34] and *ITGB3* [17q21] loci. The association of the ABO type to the development of severe NAIT could be due to a potential linkage to one or more gene(s) encoding regulatory factors. Further investigation has to be conducted to find out whether such factors are linked to the *ABO* locus in a way that can explain our observation. 

## 5. Conclusions

The development of severe NAIT in newborns is caused by transfer of platelet-reactive antibodies during pregnancy; however, several biological factors likely play a role in the immune response mechanism. In the present study, with data from a prospective NAIT study, we showed that the risk of severe NAIT due to anti-HPA-1a antibodies is correlated to maternal ABO types. The results indicate that there are genetic properties related to the maternal *ABO* genotype that influence the immune response that cause severe thrombocytopenia in the newborn of anti-HPA-1a immunized mothers.

## Figures and Tables

**Table 1:** The maternal ABO type distribution in the pregnancies compared to the severity of NAIT.

Maternal ABO type	Numbers of newborns with platelet count <50 × 10^9^/L (% within ABO type; 95% CI)	Numbers of newborns with platelet count 50–150 × 10^9^/L	Numbers of newborns with platelet count >150 × 10^9^/L	*P* value^†^
A	34 (46.6; 0.36–0.58)	13	26	0.005
O	12 (20.0; 0.12–0.32)	14	34	
B	7 (38.9; 0.20–0.61)	1	10
AB	1 (14.3; 0.03–0.51)	1	5

Total	54	29	75	

Relative risk of NAIT was 0.67 (95% CI 0.48–0.94) in neonates born of women with blood group O versus blood group A.

Relative risk of severe NAIT was 0.43 (95% CI 0.25–0.75) in neonates born of women with blood group O versus blood group A.

^†^Chi-square test (two-sided) for frequencies of NAIT and severe NAIT in blood group O compared to blood group A.

**Table 2:** Platelet counts in the newborns with NAIT.

Maternal ABO types		N*	Median platelet count	Mean platelet count (95% CI)	*P* value with Bonferroni correction
Blood group O	Genotype *O01/O01 *	9	86	83.2 (56.9–109.6)	
	Genotypes *O01/O02* and *O02/O02 *	17	21	43.7 (22.7–64.7)	0.043^†^
Blood group A		47	31	46.1 (35.0–57.2)	0.028^†^

*Total number of pregnancies resulting in a newborn with NAIT (platelet count ≤150) was 83. DNA for genotyping was available for 68 women with blood group O or A, and they had altogether 73 HPA-1-incompatible pregnancies.

^†^Comparison with platelet counts in children born of women with the genotype *O01/O01*.

## References

[1] Newman PJ, Derbes RS, Aster RH (1989). The human platelet alloantigens, PI(A1) and PI(A2), are associated with a leucine33/proline33 amino acid polymorphism in membrane glycoprotein IIIa, and are distinguishable by DNA typing. *Journal of Clinical Investigation*.

[2] Cooling LLW, Kelly K, Barton J, Hwang D, Koerner TAW, Olson JD (2005). Determinants of ABH expression on human blood platelets. *Blood*.

[3] Kjeldsen-Kragh J, Killie MK, Tomter G (2007). A screening and intervention program aimed to reduce mortality and serious morbidity associated with severe neonatal alloimmune thrombocytopenia. *Blood*.

[4] Bertrand G, Martageix C, Jallu V, Vitry F, Kaplan C (2006). Predictive value of sequential maternal anti-HPA-1a antibody concentrations for the severity of fetal alloimmune thrombocytopenia. *Journal of Thrombosis and Haemostasis*.

[5] Jægtvik S, Husebekk A, Aune B, Øian P, Dahl LB, Skogen B (2000). Neonatal alloimmune thrombocytopenia due to anti-HPA 1a antibodies; the level of maternal antibodies predicts the severity of thrombocytopenia in the newborn. *British Journal of Obstetrics and Gynaecology*.

[6] Killie MK, Husebekk A, Kjeldsen-Kragh J, Skogen B (2008). A prospective study of maternal anti-HPA 1a antibody level as a potential predictor of alloimmune thrombocytopenia in the newborn. *Haematologica*.

[7] L’Abbe D, Tremblay L, Filion M (1992). Alloimmunization to platelet antigen HPA-1a (PIA1) is strongly associated with both HLA-DRB3^*∗*^0101 and HLA-DQB1^*∗*^0201. *Human Immunology*.

[8] Williamson LM, Hackett G, Rennie J (1998). The natural history of fetomaternal alloimmunization to the platelet-specific antigen HPA-1a (PlA1, Zwa) as determined by antenatal screening. *Blood*.

[9] Killie MK, Kjeldsen-Kragh J, Randen I, Skogen B, Husebekk A (2004). Evaluation of a new flow cytometric HPA 1a screening method: a rapid and reliable tool for HPA 1a screening of blood donors and pregnant women. *Transfusion and Apheresis Science*.

[10] Skogen B, Hansen BR, Husebekk A, Havnes T, Hannestad K (1988). Minimal expression of blood group A antigen on thrombocytes from A2 individuals. *Transfusion*.

[11] Curtis BR, Fick A, Lochowicz AJ (2008). Neonatal alloimmune thrombocytopenia associated with maternal-fetal incompatibility for blood group B. *Transfusion*.

[12] Ogasawara K, Ueki J, Takenaka M, Furihata K (1993). Study on the expression of ABH antigens on platelets. *Blood*.

[13] Blumenfeld OO, Patnaik SK (2004). Allelic genes of blood group antigens: a source of human mutations and cSNPs documented in the blood group antigen gene mutation database. *Human Mutation*.

[14] Olsson ML, Chester MA (1995). A rapid and simple ABO genotype screening method using a novel B/O2 versus A/O2 discriminating nucleotide substitution at the ABO locus. *Vox Sanguinis*.

[15] Olsson ML, Chester MA (1996). Frequent occurrence of a variant O1 gene at the blood group ABO locus. *Vox Sanguinis*.

[16] Hosseini-Maaf B, Hellberg A, Chester MA, Olsson ML (2007). An extensive polymerase chain reaction-allele-specific polymorphism strategy for clinical ABO blood group genotyping that avoids potential errors caused by null, subgroup, and hybrid alleles. *Transfusion*.

[17] Hartmann O, Stavem P (1964). ABO blood-groups and cancer. *The Lancet*.

[18] Bertrand G, Drame M, Martageix C, Kaplan C (2011). Prediction of the fetal status in noninvasive management of alloimmune thrombocytopenia. *Blood*.

[19] Thuresson B, Chester MA, Storry JR, Olsson ML (2008). ABO transcript levels in peripheral blood and erythropoietic culture show different allele-related patterns independent of the CBF/NF-Y enhancer motif and multiple novel allele-specific variations in the 5’- and 3’-noncoding regions. *Transfusion*.

[20] Twu Y-C, Hsieh C-Y, Yu L-C (2006). Expression of the histo-blood group *B* gene predominates in *AB*-genotype cells. *Transfusion*.

[21] Yamamoto F, Clausen H, White T, Marken J, Hakomori S (1990). Molecular genetic basis of the histo-blood group ABO system. *Nature*.

[22] Eiz-Vesper B, Seltsam A, Blasczyk R (2005). ABO glycosyltransferases as potential source of minor histocompatibility antigens in allogeneic peripheral blood progenitor cell transplantation. *Transfusion*.

[23] Roubinet F, Despiau S, Calafell F (2004). Evolution of the O alleles of the human ABO blood group gene. *Transfusion*.

[24] Bennett EP, Steffensen R, Clausen H, Weghuis DO, van Kessel AG (1995). Genomic cloning of the human histo-blood group ABO locus. *Biochemical and Biophysical Research Communications*.

